# Continuous infusion of low-dose iohexol confirms 1-hour creatinine clearance is more accurate in acute kidney injury than 4-hour creatinine clearance: preliminary data

**DOI:** 10.1186/cc14372

**Published:** 2015-03-16

**Authors:** J Dixon, K Lane, R Dalton, I MacPhee, B Philips

**Affiliations:** 1 St George's Hospital and University of London, UK; 2 King's College, London, UK

## Introduction

There is currently no accurate method of measuring the glomerular filtration rate (GFR) during acute kidney injury (AKI). Four-hour creatinine clearance (4-CrCl) is often used. We have previously validated a method of measuring the GFR using a continuous infusion of low-dose iohexol (CILDI) in patients with stable renal function (GFR from normal to <30 ml/minute/1.73 m^2^). Steady state was achieved in <10 hours in all subjects and we calculate that variations >10.3% suggest an AKI. In this study we compare GFR measured by CILDI with 4-CrCl and 1-hour creatinine clearance (1-CrCl).

## Methods

Critically ill patients with evolving AKI and patients following nephrectomy were recruited. Demographics were compared using the *t *test. CIDLI was connected for up to 72 hours. Plasma and renal iohexol and creatinine concentrations were measured by tandem mass spectrometry four times daily. Iohexol renal clearance (IRC) and 1-CrCl and 4-CrCl were calculated and compared using Bland-Altman analysis.

## Results

Baseline estimated GFR was similar in the postnephrectomy (88 ± 28) to the evolving AKI group (92 ± 23), *P *= 0.70. The evolving AKI group had a higher APACHE score (17.8 ± 5.1 vs. 10.6 ± 3.9; *P *< 0.001). When 1-CrCl was compared with IRC, a bias of 0.8% (SD 26%, limits of agreement -52 to 50%; Pearson's *r *= 0.90) was observed in the evolving AKI group, whereas bias was -3.3% (SD 16, limits of agreement -35 to 29%; Pearson's *r *= 0.95) in the postnephrectomy group. When 4-CrCl was compared with IRC, bias was 5.1% (SD 54, limits of agreement -102 to 112%, Pearson's *r *= 0.45) in the established AKI group and bias was -4.5% (SD 38, limits of agreement -79 to 70%; Pearson's *r *= 0.78) in the postnephrectomy group.

## Conclusion

Our data suggest that 4-CrCl is not as accurate and precise as 1-CrCl in patients with AKI and following nephrectomy. IRC appears to be more accurate and precise in patients with a predicted AKI risk and outcome (post nephrectomy) than in patients with evolving AKI. We hypothesise that IRC will be useful alternative to creatinine-based measures of AKI.

